# Malaria parasitaemia, anaemia and malnutrition in children less than 15 years residing in different altitudes along the slope of Mount Cameroon: prevalence, intensity and risk factors

**DOI:** 10.1186/s12936-018-2492-1

**Published:** 2018-09-24

**Authors:** Rene Ning Teh, Irene Ule Ngole Sumbele, Derick Ndelle Meduke, Samuel Takang Ojong, Helen Kuokuo Kimbi

**Affiliations:** 1 Department of Zoology and Animal Physiology, University of Buea, Buea, Cameroon; 2 Clinical Diagnostic Laboratory, University of Buea, Buea, Cameroon; 3 Department of Medical Laboratory Sciences, University of Bamenda, Bamenda, Cameroon

**Keywords:** Malaria parasite, Anaemia, Malnutrition, Altitude, Children, Prevalence, Risk factors, Cameroon

## Abstract

**Background:**

Malaria, anaemia and malnutrition are frequently co-existing diseases that cause significant morbidity and mortality particularly among children. This study measured the prevalence, intensity and evaluated risk factors for malaria parasitaemia, anaemia and malnutrition among children living at low versus high altitude settings in the Mount Cameroon area.

**Methods:**

A cross-sectional community based survey involving 828 children aged 6 months to 14 years was conducted between July and November 2017. Malaria parasitaemia was confirmed by light microscopy, haemoglobin concentration was measured using an auto haematology analyser, nutritional status was determined from the anthropometric measurements collected, and socioeconomic status related variables by the use of questionnaire. Anaemia and malnutrition were defined according to World Health Organization standards. Associations between predictor variables and primary outcomes were assessed using logistic regression analysis.

**Results:**

Malaria parasite and anaemia were prevalent in 41.7% and 56.2% of the children, respectively while, malnutrition prevalence was 34.8% with wasting, underweight and stunting occurring in 25.7%, 19.9% and 23.7% of them respectively. Overall malaria parasite geometric mean density was 413/µL of blood (range 100–27,060). The odds of having malaria parasitaemia was highest in children 5–9 years of age [odd ratio (OR) = 1.69, P = 0.006], living in lowland (OR = 1.48, P = 0.008) as well as those whose domestic water was collected from an open source (streams/springs) (OR = 1.81, P = 0.005) than their counterparts. Being < 5 years (OR = 3.15, P = < 0.001) or 5–9 years (OR = 2.20, P < 0.001) of age, having malaria parasite (OR = 2.07, P = < 0.001) and fever in the past 2 days (OR = 1.52, P < 0.04) were identified as significant risk factors of anaemia while the age group < 5 years was the only significant risk (OR = 3.09, P = < 0.001) associated with malnutrition.

**Conclusion:**

While age specific attention should be given in the control of malaria (5–9 years), anaemia (< 10 years) and malnutrition (< 5 years), the existing malaria control programmes should be revised to integrate anaemia and malnutrition control strategies so as to improve upon the health of the children.

## Background

The proportion of the population at risk in sub-Saharan Africa who are infected with malaria parasites is estimated to have declined from 22% in 2005 to 13% in 2015, leading to a decrease, from 146 million to 114 million [1]. In Cameroon, malaria burden and transmission intensity is heterogeneous with spatial and temporal variations between altitudes and geographical areas, with prevalence rates varying from one area to another [2]. Like many sub-Saharan countries, the prevalence of malaria has dropped across the country since the implementation of the use of insecticide-treated nets (ITN) in 2007 [3–6]. A follow up study carried out by Sumbele et al. [3] in the Mount Cameroon area between 2006 and 2013 showed that the prevalence of malaria parasitaemia dropped from 85.4% in 2006 to 36.6% in 2013 with a relative risk reduction of 57.2%. Nevertheless, malaria still remains a major killer of children in this country, and is estimated to take the life of a child every 2 min [1].

Malaria, anaemia and under-nutrition are each associated with significant morbidity and mortality, with higher rates among children particularly in sub-Saharan Africa [7–9]. Anaemia is a condition where due to low blood haemoglobin concentration [7] the oxygen carrying capacity of red cells is insufficient to meet the body’s physiologic needs. This condition affects individuals globally and has significant adverse health consequences, as well as adverse impacts on social and economic development [10]. Childhood anaemia is considered a severe public health problem in Sub-Saharan Africa (62.5%) and in Cameroon in particular where prevalence of 63.2% was reported in 2011 [1]. Malaria causes a substantial proportion of anaemia observed in malaria endemic settings [11–13]. Notwithstanding, updating the role of malaria parasitaemia on anaemia in an era where the coverage of ITN is above 75% in Cameroon [14] will help the National Malaria Control Programmes to plan proper management strategies, while taking into consideration the levels of heterogeneities that exists within different localities. Even so, how much of the anaemia burden is associated with malaria, relative to other causes such as malnutrition, and its variation across the different altitudes of the Mount Cameroon area has not been established.

Nutritional status is closely tied to immune responses to infection, being on the one hand, an important determinant of the risk and prognosis of infectious diseases, and on the other hand, being directly influenced by infection [15]. To date, findings from studies evaluating associations between various measures of malnutrition and malaria have been contradictory. Sumbele et al. [16] reported that malnutrition is associated with a higher risk of *Plasmodium* infection and infectious episodes contribute to the deterioration of nutritional status. In contrast, some studies found no association between nutrition and subsequent mortality from malaria [9, 17]. Yet, malnutrition and *Plasmodium falciparum* malaria frequently co-exist in Sahelian countries and account for a large part of under-five morbidity and mortality during their concomitant peak seasons [18].

Based on the 2013 United Nations Children’s Fund report, 38% of children below the age of 5 years suffer from chronic malnutrition or stunting in sub-Saharan Africa, with malaria and under nutrition being the two major causes of childhood mortality [19]. Anaemia has also been reported as a significant determinant of stunting [20], which is the main type of malnutrition in young children [21]. Stunting is associated with impaired cognitive development, reduced academic achievement, and decreased physical work capacity in adulthood, with negative cost on economic development of societies [22]. While the global stunting prevalence fell from 39.6 to 23.8% between 1990 and 2014, the scenario is quite different in Africa, with an increase [23]. Nevertheless, in some localities in the Mount Cameroon area the prevalence of stunting fell from 49.9% [24] to 17.1% [21]. The impact of nutritional status on malaria may differ due to the heterogeneity of the population under study, species of the parasite, and other factors involved in host and parasite relationship. The study aimed at determining the prevalence and intensity of malaria parasitaemia, anaemia and malnutrition as well as identifying the risk factors for these public health concerns among children living in low versus high altitude settings in the Mount Cameroon area.

## Methods

### Study sites and participants

The study was carried out in Batoke (Limbe) and Tole (Buea), which are two different altitudinal ranges along the slope of the Mount Cameroon area. The sites were classified as lowlands [< 200 m above sea level (asl)] and highlands (> 600 m asl). The coordinates of Batoke ranged from altitude 8 m, latitude 04°01.364′N, longitude 009°05.971′E to 47 m, 04°02.039′N and009°05.808′E. Tole is located between 627 m, latitude 04°07.057′N, longitude 009°15.178′E and 630 m, latitude 04°6.906′N, longitude 09°14.434′E. The Mount Cameroon area stretches from the Atlantic Ocean at the Gulf of Guinea, gradually increasing from Limbe to 800–1200 meters in Buea on the eastern flank of the Mountain. The temperatures in lowlands (Limbe) are high and fairly constant ranging between 25 and 30 °C on average [25] with abundant rainfall ranging from 5500 to 6500 mm per annum. In the highlands (Buea), weather records from the Cameroon Development Corporation indicate a mean relative humidity of 80%, an average rainfall of 4000 mm and a temperature range of 18–27 °C [26]. The Tole study area has been described in detail by Ndamukong et al. [27]. The Mount Cameroon study area is subjected to a Cameroonian-type equatorial climate characterized by fairly constant temperatures and two seasons: a short dry season (November–February) and a long rainy season (March–October) with abundant precipitation (2000–10,000 mm) [28]. Malaria transmission is perennial with two peaks in season, the first between April and May and the second between October and November. These correspond with the beginning and end of the rainy season, respectively. *Plasmodium falciparum* is also the main species and *Anopheles gambiae* is the main vector species [29].

The study was carried out among pre and school age children of both sexes aged 6 months to 14 years old, weighed > 5 kg, free from other clinical conditions not related to malaria and sickle test negative. Children with severe malaria (unable to drink or breastfeed, vomiting more than twice in the preceding 24 h before presentation, recent history of convulsions, unconscious state or unable to sit or stand and other diseases requiring hospital admission) were excluded from the study.

### Study design

This cross sectional community based study was carried out between the months of July and November 2017, reported as the peak malaria transmission period in the Mount Cameroon area [26]. Ensuing administrative clearances and ethical approval for the study, informed consent/assent forms explaining the purpose, risks, and benefits of the study were given to parent/caregivers. Participants were invited to the data collection location in each community by their local chiefs and coordination was organized by the head/leader of a block within a neighbourhood (quarter head) of the various communities. Upon obtaining consent/assent from the parents/care givers, the study team proceeded for sample collection. Following administration of a semi-structured questionnaire, body temperature, anthropometric measurements and blood sample were collected from each child for malaria parasite identification and a full blood count assessment. The sample size for each study altitude was calculated using the 66.2% prevalence of malaria in children in the study area [27]. Sample size was determined using the formula n = Z^2^pq/d^2^ [30] where n = the sample size required, z = 1.96: which is the standard normal deviate (for a 95% confidence interval, CI), p = 66.2%: proportion of malaria prevalence, q = 1 − p: proportion of malaria negative children and d = acceptable error willing to be committed. The minimum sample size was estimated as n = 344 for each site. Considering a possible participation of more than one child per family, loss of samples due to blood clotting and incomplete data entry, the sample size was adjusted by 10% to a minimum of 379. Following education of the community for each family to ensure the participation of at most a child less than 15 years in the study a date for collection of sample was set. Potential participants were reminded of the collection dates per block by the head/leader of the block. A convenience sampling method was used in all the blocks in each altitude until the required sample was attained. At the start of the study in each site, the parents, guardians and children were educated on the study protocol and the benefits of participation highlighted at their various neighbourhoods.

### Clinical evaluation

The axillary temperature was measured using a digital thermometer and fever was defined as temperature ≥ 37.5 °C [12]. Anthropometric measurements such as height and weight were measured using a measuring tape and a Terraillon weighing scale (Terraillon, Paris), respectively. To ensure the accuracy of using a tape to measure the height of ambulant children, the tape was attached to a locally constructed wood work that served as a stadiometer. Under-nutrition indices such height-for age (HA), weight-for-age (WA), and weight-for-height (WH) standard deviation (SD) scores (Z scores) were computed based on the WHO growth reference curves using the WHO AnthroPlus for personal computers manual [31]. A child was identified as being malnourished if he or she scored < − 2 in one of the anthropometric indices of HA (stunting), WA (underweight) and WH (wasting) indices, while corresponding Z scores of < − 3 SD were considered indicative of severe under-nutrition [9].

### Questionnaire survey

A semi-structured questionnaire was administered to the child’s parent/caregiver to collect data on (i) demographics (sex, age, literacy, occupation and marital status); (ii) Socioeconomic status related variables (number of house occupants, house type, toilet type, and water sources) [26] and knowledge of malaria including sign/symptoms, complications, transmission and prevention methods (iii) fever management practices; and (iv) malaria prevention practices bed net (long lasting insecticide net (LLIN) ownership, physical integrity, number and use) and indoor residual spraying (IRS) experience. LLIN use was defined as having slept under a LLIN the night prior to the survey and the physical integrity of LLIN was assessed by checking for holes in the nets and counting them using the WHO Pesticide Evaluation Scheme [32].

### Laboratory methods

Four millilitres of venous blood samples were collected from the children using sterile disposable syringes. The collected blood was aliquot into labelled ethylenediaminetetraacetate (EDTA) tubes and the remaining blood dispensed on slides for the preparation of thick and thin blood films. Labelled blood samples in EDTA tubes were transported on ice in a cool box to the Malaria Research Laboratory, University of Buea for a full blood count analysis. Thin blood films were fixed with absolute methanol and later stained along with thick blood films using 10% Giemsa for 20 min. The films were examined following standard procedure for the detection and identification of malaria parasites [33]. As a quality control measure, slides were read by two independent parasitologists, and in the case of any disparity they were read by a third parasitologist. Slides were considered positive when asexual forms and/or gametocytes of any *Plasmodium* species were observed on the blood film. The number of parasite were counted per 200 leukocytes on thick blood film and asexual parasite densities per μL of blood was obtained by multiplying the parasite count with the participants’ white blood cell count obtained from the complete blood count analysis. If gametocytes were seen, the count was extended to 500 leukocytes. Parasitaemia was categorized as low (< 1000 parasites/μL blood), moderate (1000–4999 parasites/μL blood), high (5000–99,999 parasites/μL blood), and hyperparasitemia (≥ 100,000 μL) [3].

Haematological parameters were assessed using an auto-haematology analyser (MINRAY 2800 BC), following the manufacturer’s instructions. A complete blood count was obtained and anaemia was defined as Hb < 11.0 g/dL [27] and further categorized as severe (Hb < 7.0 g/dL), moderate (Hb between 7.0 and 10.0 g/dL), and mild (> 10 Hb < 11 g/dL) as reported by Cheesbrough [34]. Malarial anaemia (MA) was defined as children with a malaria-positive smear for *P. falciparum* parasitaemia (of any density) and Hb < 11 g/dL. Non-malarial anaemia was defined as children with anaemia and without a malaria-positive smear for *P. falciparum.*

### Statistical analysis

Data collected was cleaned up and analysed using the IBM-Statistical Package for Social Sciences (IBM-SPSS) version 20 and Epi-info version 7. Continuous variables were summarized into means and standard deviations and categorical variables reported as frequencies and percentages were used to evaluate the descriptive statistics. The differences in proportions were evaluated using Pearson’s Chi Square (χ^2^). Group means were compared using analysis of variance (ANOVA), Student’s t test and non-parametric tests such Mann-Withney U test and Kruskal–Wallis test where appropriate. Parasite density was log transformed before analysis. Associations between predictor variables and primary outcomes were assessed using both bivariate and multivariate logistic regression analysis. Multi-collinearity test was performed for all potentially correlated variables and only variables with variance inflation factors less 2 were included in the models. Odd ratios (ORs) and 95% confidence intervals (CIs) were computed. Any covariate with a P value < 0.2 in the bivariate analysis was subsequently included in the final multivariable logistic model. Significant levels were measured at 95% CI with the level of significance set at P < 0.05.

## Results

### Characteristics of study participants

The socio-demographic and clinical characteristics of the study participants are shown in Table 1. A total of 828 children with a mean (SD) age of 6.5 (3.6) years, residing at lowland (48.9%, 405) and highland (51.1%, 423) in the Mount Cameroon area were evaluated. There was a slight majority of females (52.4%) than males (47.6%) although not significant. Most of the study participants were in the age group 5–9 years (41.3%) and the least in the age group 10–14 years (23.8%). A greater proportion of the parents/guardians of the children had a primary level of education (52.2%) followed by those having secondary school education (35.6%).

**Table 1:** Socio-demographic and clinical characteristics of study population

Parameter	Total
% (N)	100 (828)
Sex	
Female	52.4 (434)
Male	47.6 (394)
Age groups in years	
< 5	34.9 (289)
5–9	41.3 (342)
10–14	23.8 (197)
Educational level of parent/caregiver	
No formal (n)	5.9 (44)
Primary (n)	52.2 (392)
Secondary (n)	35.6 (267)
Tertiary (n)	6.4 (48)
Altitude of residence	
Highland (n)	51.1 (423)
Lowland (n)	48.9 (405)
LLIN use	
Yes	49.9 (413)
No	50.1 (415)
Clinical	
Mean age (SD) in years	6.5 (3.6)
Mean weight (SD) in kg	24.9 (19.5)
Mean height (SD) in cm	111.7 (23.5)
Mean temperature (SD) in °C	36.7 (0.7)
Fever prevalence (n)	9.5 (79)
Malaria parasite prevalence (n)	41.7 (345)
Mean haemoglobin level (g/dL)	10.7 (0.7)
Anaemia prevalence (n)	56.2 (465)
Malarial anaemia prevalence (n)	27.7 (201)
Non malaria anaemia prevalence (n)	28.5 (236)
Malnutrition (n)	34.8 (288)
Wasting (n)	25.7 (87)^a^
Underweight (n)	19.9 (136)^b^
Stunting (n)	23.7 (196)

^a^Wasting was evaluated for 338 participants ≤ 5 years

^b^Underweight was evaluated for 683 participants ≤ 10 years

The proportion of children who slept under a mosquito net the previous night of the study was (49.9%). Fever, malaria parasite, anaemia and malnutrition were observed in 9.5% (79), 41.7% (345), 56.2% (465) and 34.8% of the children, respectively.

### *Plasmodium* prevalence, density and predictors

The prevalence of falciparum malaria among the 828 children varied with altitude. Children of the lowlands had a significantly higher (P = 0.004) prevalence of malaria parasite (46.7%) than those of the highlands (36.9%), as shown in Table 2. Similarly, the geometric mean parasite density (GMPD) was higher in children of the lowland than their highland counterparts, although the difference was not significant. Malaria parasite prevalence was comparable between males (40.1%) and females (43.1%). A significant difference (P = 0.018) was observed with age, with the 5–9 years age group having the highest prevalence (46.8%) followed by the < 5 years age group (40.5%) and least, the 10–14 age group.

**Table 2:** Malaria parasite prevalence and density with respect to altitude, sex and age

Parameter	No. examined	Prevalence (n)	P value	GMPD (SD)/µL of blood	Range/µL of blood)	P-value
Altitude						
Low land	405	46.7 (189)	0.004*	449 (1894.5)	100–11,520	0.26^a^
Highland	423	36.9 (156)		374 (3253.8)	102–27,060	
Gender						
Male	394	40.1 (158)	0.384	400 (1664.3)	100–10,920	0.587^a^
Female	434	43.1 (187)		424.5 (3196.2)	102–27,060	
Age group in years						
< 5	289	40.5 (117)	0.018*	469 (2996.8)	104–27,060	0.032*^b^
5–9	342	46.8 (160)		420 (2722.2)	100–25,546	
10–14	197	34.5 (68)		320 (1216.6)	100–57,040	
Total	828	41.7 (345)		413 (2601.8)	100–27,060	

* Statistically significant P value

^a^Difference in GMPD in the different altitude and sex determined by Mann–Whitney *U* test

^b^Difference in GMPD in the different age groups determined by Kruskal–Wallis test

**Fig. 1:**
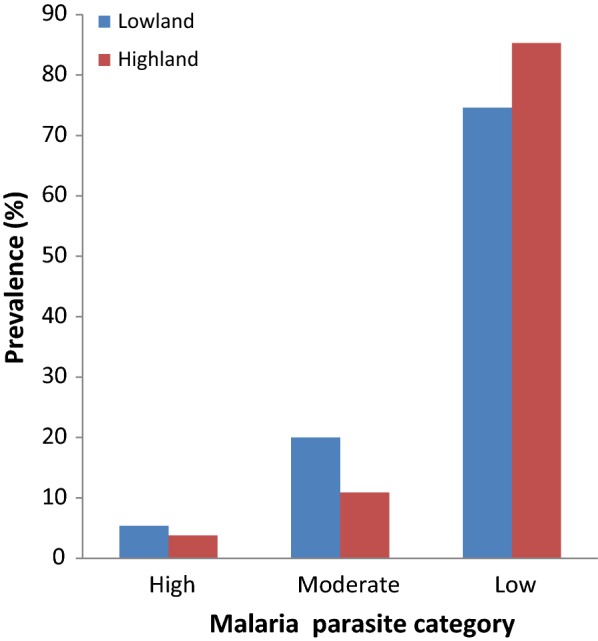
Malaria parasite density category as affected by altitude

As shown in Fig. 1, the prevalence of low and moderate malaria parasitaemia was marginally significant (P = 0.048) in children from low altitude when compared with their high-altitude counterparts and the low parasite density category was the most common in both settings.

A logistic regression model demonstrated altitude (P = 0.008), age groups (P = 0.006) and water source (P = 0.005) as significant predictors of malaria parasite prevalence as shown in Table 3. Children of the lowland were 1.48 times more likely to suffer from malaria than those of the highland. Children in the age group 5–9 years were significantly associated with a 1.69 fold higher odds of infection than their contemporaries. In addition, children from households where the domestic water was collected from an open source (streams/springs) were 1.81 fold more likely to have *Plasmodium* infection when compared with those whose domestic water was collected from a close source (tap and borehole).

**Table 3:** Logistic regression model examining factors associated with malaria parasite in children

Variables	N	Malaria parasite prevalence (n)	Bivariate logistic regression		Multivariate logistic regression	
			COR (95% CI)	P value	AOR	P value
Altitude						
Highland	423	36.9 (156)	Reference		Reference	
Lowland	405	46.7 (189)	1.48 (1.13–1.98)	0.004*	1.48 (1.12–1.96)	0.008*
Age group (Years)						
10–14	197	34.5 (68)	Reference		Reference	
5–9	342	46.8 (160)	1.67 (1.16–2.40)	0.007*	1.69 (1.17–2.44)	0.006*
< 5	289	40.5 (117)	1.24 (0.68–1.47)	0.153	1.26 (0.86–1.85)	0.24
Gender						
Female	434	43.1 (187)	Reference	–	–	–
Male	394	40.1 (158)	0.88 (0.67–1.11)	0.384	–	–
Marital status						
Married	610	42.1 (257)	Reference	–	–	–
Single	202	40.6 (82)	1.07 (0.77–1.47)	0.702	–	–
Use of LLINs						
No	415	42.4 (176)	Reference	–	–	–
Yes	413	40.9 (169)	1.06 (0.11–1.40)	0.543	–	–
Malnourished						
No	540	42.4 (229)	Reference	–	–	–
Yes	288	40.3 (116)	0.9 (0.68–1.23)	0.555	–	–
Water source						
Close	721	39.7 (286)	Reference	–	Reference	–
Open	105	55.2 (58)	1.88 (1.24–2.84)	0.002*	1.81 (1.19–2.75)	0.005*
Wasted						
No	251	41.4 (104)	Reference	–	–	–
Yes	87	39.1 (34)	1.42 (0.51–4.02)	0.50	–	–
Underweight						
No	547	43.3 (237)	Reference	–	–	–
Yes	136	43.4 (59)	0.99 (0.45–2.20)	0.99	–	–
Stunted						
No	632	41.8 (264)	Reference		–	–
Yes	196	41.3 (81)	0.77 (0.44–1.35)	0.37	–	–

* Statistically significant P value, *CI* confidence interval, *COR* crude odd ratio, *AOR* adjusted odd ratio

### Anaemia prevalence, severity and risk factors

The prevalence of anaemia was significantly higher (P < 0.001) in the youngest age group (66.1%) than those older and in children who were malaria parasite positive (66.4%) than those negative as shown in Table 4. Anaemia prevalence was however comparable in children of the different altitudes, sexes and nutritional status.

**Table 4:** Anaemia prevalence and severity in the study population

Parameter		No. examined	Prevalence (%) of anaemia (n)	No. examined	Anaemia severity		
					Severe % (n)	Moderate % (n)	Mild % (n)
Altitude	Lowland	405	54.6 (221)	221	0.9 (2)	6.3 (14)	92.8 (205)
	Highland	423	57.7 (224)	244	2.5 (6)	14.8 (36)	82.8 (202)
Level of significance	χ^2^ = 0.82, P = 0.37			χ^2^ = 10.59, P = 0.005*			
Sex	Male	394	56.9 (224)	224	2.2 (5)	10.3 (23)	87.5 (196)
	Female	434	55.5 (241)	241	1.2 (3)	11.2 (27)	87.6 (211)
Level of significance	χ^2^ = 0.15, P = 0.70			χ^2^ = 0.75, P = 0.69			
Age group in years	< 5	289	66.1 (191)	191	2.6 (5)	14.1 (27)	83.2 (159)
	5–9	342	57.9 (198)	198	1 (2)	9.1 (18)	89.9 (178)
	10–14	197	38.6 (76)	76	1.3 (1)	6.6 (5)	92.1 (70)
Level of significance	χ^2^ = 36.73 P < 0.001*			χ^2^ = 6.03, P = 0.20			
Asexual parasite Status	Negative	483	48.9 (236)	236	1.7 (4)	11 (26)	87.3 (206)
	Positive	345	66.4 (229)	229	1.7 (4)	10.5 (24)	87.8 (206)
Level of significance	χ^2^ = 25.08 P < 0.001*			χ^2^ = 0.04 P = 0.98			
Malnutrition	No	540	54.4 (294)	294	2 (6)	9.9 (29)	88.1 (259)
	Yes	288	59.4 (171)	171	1.2 (2)	12.3 (21)	86.5 (148)
Level of significance	χ^2^ = 1.86 P = 0.17			χ^2^ = 1.09, P = 0.58			
Stunting	No	632	54.6 (345)	345	1.7 (6)	9.9 (34)	88.4 (305)
	Yes	196	61.2 (120)	120	1.7 (2)	13.3 (16)	85 (102)
Level of significance	χ^2^ = 2.68 P = 0.10			χ^2^ = 1.12 P = 0.57			

* Statistically significant P value, *CI* confidence Interval, *COR* crude odd ratio, *AOR* adjusted odd ratio

Relating to anaemia severity, children in highland had a higher prevalence of severe (2.5%) and moderate anaemia (14.8%) than children in lowland (0.9% and 6.3%, respectively) and the difference was statistically significant at P = 0.005.

As shown in Table 4, the prevalence of moderate and mild anaemia was comparable in males and females. Although the difference was not statistically significant, the youngest age group had the highest prevalence in severe (2.6%) and moderate anaemia (14.1%) when compared with the older age groups while, malnourished children suffered more with moderate anaemia (12.3%) when compared with healthy children.

**Fig. 2:**
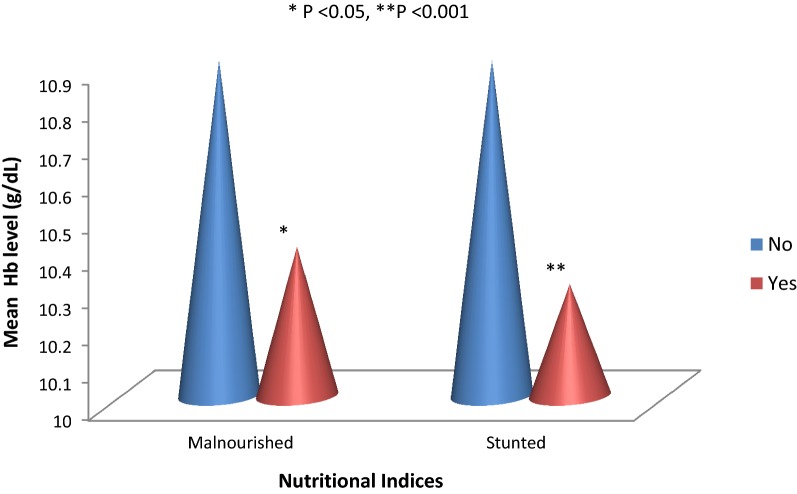
Mean Hb profile with respect to malnutrition and stunting

As shown on Fig. 2, malnourished children had significantly lower mean haemoglobin level when compared with well-nourished children (P < 0.008). In addition, stunted children had a significantly lower mean haemoglobin level when compared with those without stunting (P < 0.001).

The logistic regression model with anaemia status as dependent variable and altitude, age, gender, level of education and marital status of guardian, nutritional status, malaria status as well as fever history as the independent variables revealed the age group (P = < 0.001) malaria status (P = < 0.001) and fever in the past 2 days (P < 0.04) as significant risk factors of anaemia as shown in Table 5. Children < 5 years of age, those between 5 and 9 years, malaria parasite positive and those who had fever in the past 2 days were 3, 2, 2 and 1.52 times, respectively more likely to be anaemic than their counterparts.

**Table 5:** Factors influencing the prevalence of anaemia in the study population

Variables	N	Prevalence of anaemia (n)	Bivariate logistic regression		Multivariate logistic regression	
			COR (95% CI)	P value	AOR (95% CI)	P value
Altitude						
Highland	423	57.7 (224)	Reference			
Lowland	405	54.6 (221)	0.88 (0.67–1.16)	0.37	–	–
Age group (years)						
10–14	197	38.6 (76)	Reference		Reference	
5–9	342	57.9 (198)	2.20 (1.54–3.16)	< 0.001*	2.18 (1.50–3.17)	< 0.001*
< 5	289	66.1 (191)	3.07 (2.11–4.47)	< 0.001*	3.15 (2.11–4.70)	< 0.001*
Gender						
Female	434	55.5 (241)	Reference			
Male	394	56.9 (224)	1.01 (0.80–1.39)	0.70	–	–
Level of education						
No formal	44	56.8 (25)	Reference			
Primary	292	58.4 (229)	1.07 (0.57–2.00)	0.84	–	–
Secondary	267	55.4 (148)	0.95 (0.49–1.79)	0.86	–	–
Tertiary	48	47.9 (23)	0.69 (0.31–1.59)	0.39	–	–
Marital status						
Married	202	55.4 (112)	Reference			
Single	610	56.9 (347)	1.06 (0.77–1.46)	0.72	–	–
Malnourished						
No	540	54.4 (294)	Reference		Reference	
Yes	288	59.4 (171)	1.22 (0.92–1.63)	0.17	1.07 (0.78–1.49)	0.66
Wasted						
No	251	68.5 (172)	Reference			
Yes	87	59.8 (52)	0.98 (0.36–2.69)	0.97	–	–
Underweight						
No	547	59.8 (327)	Reference			
Yes	136	57.4 (78)	0.81 (0.44–1.51)	0.51	–	–
Stunted						
No	632	54.6 (345)	Reference			
Yes	196	61.2 (120)	0.79 (0.53–1.17)	0.23	–	–
Malaria status						
No	483	48.9 (236)	Reference		Reference	
Yes	345	66.4 (229)	2.07 (1.55–2.75)	< 0.001*	2.07 (1.53–2.79)	< 0.001*
Fever in the past 2 days						
No	650	54.2 (352)	Reference		Reference	
Yes	155	63.2 (98)	1.44 (1.01–2.06)	0.04*	1.52 (1.01–2.11)	0.04*

* Statistically significant P value, *CI* confidence Interval, *COR* crude odd ratio, *AOR* adjusted odd ratio

### Malnutrition in the study population

**Fig. 3:**
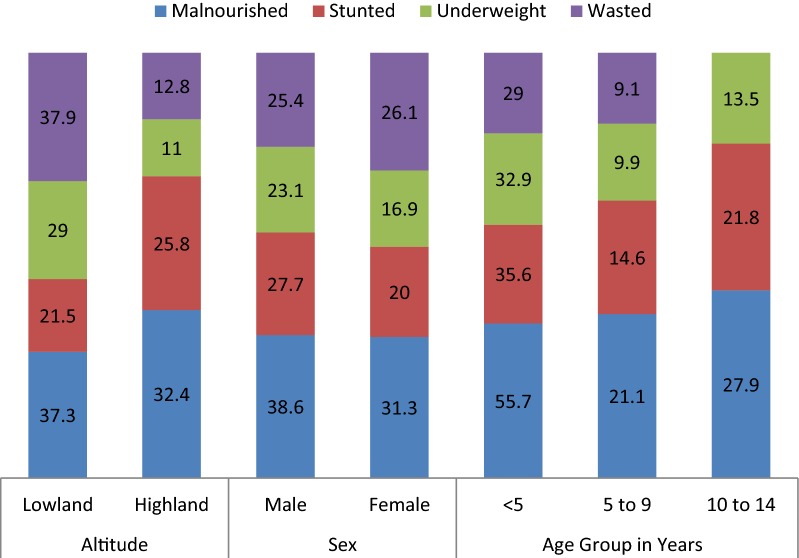
Effect of altitude, sex and age on the prevalence of malnutrition and its forms

As shown in Fig. 3, the prevalence of malnutrition was higher in children of the lowland (37.3%) than highland (32.4%) although not statistically significant. However, more males were malnourished (38.6%) when compared with females (31.3%) and the difference was significant at P = 0.03. While stunting (P = 0.01) and underweight (P = 0.04) were significantly higher in males, the difference in prevalence of wasting among the sexes was not significant (P = 0.89).

The prevalence of malnutrition varied significantly (P < 0.001) with the age group. The highest prevalence was observed in children of the < 5 years age group (55.7%) and lowest in children 5–9 years old (21.1%). Similarly, the prevalence of stunting (35.6%), underweight (32.9%) and wasting (29.0%) was higher in the under-five age group compared to the older children (Fig. 3).

A bivariate analysis revealed being male (P = 0.004) and children of the < 5 years age group (P < 0.001) were significantly at odds of being malnourished. However, in the final multivariable model, age was the only significant predictor where children under 5 years of age were three times more likely to be malnourished than those older as shown in Table 6.

**Table 6:** Logistic regression model showing risk factors of malnutrition

Variables	N	Prevalence of malnutrition (n)	Bivariate logistic regression		Multivariate logistic regression	
			COR (95% CI)	P value	AOR	P value
Altitude						
Highland	423	32.4 (137)	Reference		Reference	
Lowland	405	37.3 (151)	1.24 (0.93–1.65)	0. 14	1.18 (0.87–1.59)	0.30
Age group						
10–14	197	27.9 (55)	Reference		Reference	
5–9	342	21.1 (72)	0.69 (0.46–1.04)	0.07	0.68 (0.45–1.03)	0.07
< 5	289	55.7 (161)	3.22 (2.18–4.75)	< 0.001*	3.09 (2.08–4.6)	< 0.001*
Gender						
Female	434	31.3 (136)	Reference		Reference	
Male	394	38.6 (152)	1.38 (1.03–1.83)	0.0291*	1.22 (0.90–1.65)	0.20
Level of education						
No formal	44	29.5 (13)	Reference			
Primary	392	33.2 (130)	1.18 (0.59–2.34)	0.63	–	–
Secondary	267	36.7 (98)	1.38 (0.69–2.77)	0.35	–	–
Tertiary	48	43.8 (21)	1.85 (0.78–4.40)	0.16	–	–
Marital status of parent/guardian						
Married	611	34.5 (211)	Reference			
Single	201	37.8 (76)	1.08 (0.77–1.50)	0.66	–	–
Fever in the past 2 days						
No	650	35.4 (230)	Reference			
Yes	155	31.6 (49)	0.85 (0.59–1.24)	0.41	–	–
Malaria status						
Negative	483	35.6 (172)	Reference			
Positive	345	33.6 (116)	0.87 (0.63–1.19)	0.38	–	–
Anaemia status						
Negative	363	32.2 (117)	Reference		Reference	
Positive	465	36.8 (171)	1.22 (0.92–1.63)	0.17	1.06 (0.77–1.45)	0.73

* Statistically significant P value, *CI* confidence Interval, *COR* crude odd ratio, *AOR* adjusted odd ratio

## Discussion

This cross-sectional study examines *P. falciparum* malaria, anaemia, and malnutrition as public health problems in children < 15 years across low and highland altitudes in the Mount Cameroon area. The overall malaria parasitaemia of 41.7% observed by microscopy in the study population reveals malaria remains a major cause of illness during childhood. The observation is similar to that reported by Lehman et al. [35] in schoolchildren from the Littoral Region of Cameroon. However, the prevalence is lower than the 66.9% reported earlier in children ≤ 14 in Tole community [27] and the 45.3% in pupils between 4 and 16 years in other areas in the Mount Cameroon area [2]. Even though a lower prevalence has been reported by Apinjoh et al. [5] and Nyasa et al. [36] in the Mount Cameroon area, the GMPD observed in the study (413 parasites/µL of blood) is lower than the 1721 parasites/µL of blood reported by Apinjoh et al. [5]. Although, the Mount Cameroon area has an equatorial climate characterized by abundant rainfall and constant humidity which are factors favouring intense and perennial transmission of the malaria parasite [37], the decrease in malaria morbidity is thought to be the result of sustained control measures including implementation of long lasting insecticide nets and use of artemisinin-based combination therapy (ACT) as recommended by the World Health Organization.

In line with Kimbi et al. [26] and Ndamukong-Nyanga et al. [38], malaria prevalence was higher in children living in lower altitudes than their higher altitude counterparts. Other studies have also reported a drop in malaria prevalence from lowland to highland altitude in the Mount Cameroon area [29]. This is not atypical as minimum and maximum temperatures drop by 1 °C after every 100 m rise in altitude hence, this conditions becomes less favourable for the mosquito vector which is known to thrive more in warmer climates. Worthy of note is that, while the prevalence of malaria parasite obtained in the lowland (46.7%) is lower than the 60.5% obtained in earlier studies in the same Mount Cameroon area [38], that of the highland demonstrated rather an increase from 7.7% [26] or 15.4% [38] to 36.9%. The continuous increase in prevalence of malaria parasite in highland communities when a decrease is observed in the lowland, probably demonstrate the changing environmental conditions such as temperature and anthropogenic activities which provides favourable micro climatic conditions for the mosquito vector to thrive. In addition, higher temperatures also favour the *Plasmodium* to complete its sporogonic cycle within a shorter time in the mosquito vector [39].

Findings from the study revealed the odds of having malaria was highest in the 5–9 years age group. The epidemiological shift in malaria burden from the under-five age group to the 5–9 years age group is in line with previous surveys in this part of the country [5, 6, 40]. This could probably be due to the intensive malaria control including free ITN and ACT for the less than five age group in all government health centres in the country. A decrease in malaria exposure due to proper usage of ITN for the under-five age group could plausibly impede or delay development of malaria protective immunity leading to an increased odds of malaria in the 5–9 years age groups. Also, it is unlikely that maternal care has reduced in this age group (< 5 years) as the child becomes independent and less likely to use the ITN. Consequently, health education and treatment should not only target vulnerable groups (children under 5 and pregnant women), but all the age groups. However, similar to findings by Ndamukong et al. [38], the 10–14 years age group recorded the lowest malaria prevalence and parasite load. Children in this age group are likely to acquire protective immunity, after repeated exposure to malaria infection [3].

In line with previous study in Rwanda [9], living in houses where domestic water was sourced from an open source (streams and springs) compared to households where domestic water was drawn from a closed source (tap and borehole) was associated with a high odds of malaria infection. Regarding the domestic water sources, open water sources may also serve as potential mosquito breeding sites and hence pose an increased risk [9].

The high prevalence of overall anaemia in children less than 15 years highlights the impact of anaemia among the population in this area. The relationship between malaria parasitaemia and anaemia is well established in previous studies [5, 9, 12, 37]. Malaria parasitaemia causes more destruction of parasitized and non-parasitized red blood cells hence reducing haemoglobin levels leading to anaemia. Findings from the study indicated malaria positive children were twofold more likely to be anaemic, when compared with their negative counterparts. The higher prevalence of anaemia from this study compared to the 37% of Sowunmi et al. [41] from Nigeria and its association with malaria strongly suggest that malaria accounts for a major part of the burden of anaemia in this community. The higher prevalence of anaemia in the younger age group is in line with previous studies that anaemia due to malaria is more severe in younger children in areas of intense transmission [3, 42]. Children in this age group are more vulnerable to infection with malaria than others with severe and potentially fatal complications.

Interestingly, in this study, children with fever for the past 2 days were 1.52 times more likely to have anaemia. A study carried by Sumbele et al. [12] in the same study area revealed that febrile children were two times at odds of being moderate to severely anaemic than afebrile children. The fever associated anaemia could be indicative of other undetectable anaemia causing infections and not necessarily malaria which probably accounted for the 28.5% cases of non-malaria anaemia. However, data on helminthic infection could not be collected because children in this community had been de-wormed following the regular de-worming campaigns organized by the Cameroon’s Ministry of Public Health targeting mainly children.

A lower haemoglobin level, but not anaemia, was significantly associated with malnutrition and particularly stunting in the study. Evidence for the impact of under-nutrition on development of anaemia in young children living in malaria-endemic areas had been reported previously [9]. Although this study did not assess for other causal factors associated with anaemia, it is plausible that children who were stunted were more likely to also have micronutrient deficiencies that may have partly contributed to the lower haemoglobin levels compared to their non-stunted counterparts.

Malnutrition was common (34.8%) in the community with an overall prevalence of 23.7% for stunting, the most common form of malnutrition. The prevalence of stunting was lower than the 42.9% obtained by Akiyama et al. [20] in Loa People’s Democratic Republic and 30.0% by Magalhães et al. [43] in the Northern part of Angola. However, lower prevalence of 17.1% was obtained by Sumbele et al. [21] in children of Muea community in the same Mount Cameroon region and 19.4% by Nyaaba et al. [17] in Ghana. The common occurrence of this condition in a community were majority of the inhabitants are farmers is remarkable. Males were 1.2 times more likely to be malnourished than females. More specifically, stunting and underweight were significantly higher in males than females. This observation corroborates with studies carried out in other localities [9, 21].

In line with previous findings [21, 44], the prevalence of malnutrition was highest in the under-five age group than their older counterparts. Children in this age group were three times more likely to be malnourished than the oldest age group. It has also been reported that under nutrition weakens the immune system exposing the child to diseases like diarrhoea, measles and respiratory infections [19, 45]. However, concerning the relationship between malaria and malnutrition, the results are conflicting. In line with studies from the Democratic Republic of Congo [46], we observed lower odds of malaria parasitaemia among children with malnutrition. On the contrary, Gari et al. [47], from Ghana, reported malaria as a risk factor for malnutrition. The absence of association in this study could perhaps be attributed to the difference in the definition of a malaria case. Unlike other studies in which malaria was defined based on the presence of clinical features such as fever or a history of it in association with parasitaemia, this study examined the presence of malaria parasitaemia and fever as independent risk factors.

The study had as limitations some unmeasured factors such as micronutrient deficiency and markers of inflammation which may have acted as confounders on the risk of the presence of anaemia. Never the less, the findings of the study demonstrated the main factors associated with the presence of the public health problems of malaria parasitaemia, anaemia and malnutrition.

## Conclusions

While malaria, anaemia and malnutrition are still of public health concern in the Mount Cameroon area, there is a strong association between malaria and anaemia but not malnutrition. Even with the decline in morbidity (GMPD), the prevalence of falciparum malaria is highest among the 5–9 years age group when compared with the under-five or 10–14 years age group. Therefore, in the strategic planning and design of malaria control programmes due attention should be given to children residing in lowland, those whose domestic water is sourced from an open source and children in the age group 5–9 years. For the control of anaemia consideration should be directed to the younger age group (< 5 and 5–9 years), those with malaria parasitaemia, and children with a history of fever within the past 2 days. Despite the fact that stunting is the most common form of malnutrition, with males being more malnourished than females the insidious impact of stunting resulting to lower haemoglobin levels than in healthy children warrants thoughtfulness in planning its control. Hence the health of children in malaria meso-endemic areas can be improved upon if the existing malaria control programmes are revised to integrate anaemia and malnutrition control strategies.

## Abbreviations

AOR: adjusted odd ratio
asl: above sea level
ACT: artemisinin-based combination therapy
CI: confidence interval
COR: crude odd ratio
EDTA: ethylenediaminetetraacetate
GMPD: geometric mean parasite density
Hb: haemoglobin
HA: height-for age
ITN: insecticide-treated nets
LLIN: long-lasting insecticidal net
MA: malarial anaemia
OR: odd ratio
SD: standard deviation
WA: weight-for-age
WH: weight-for-height
